# miRNA deregulation targets specific pathways in leiomyosarcoma development: an in silico analysis

**DOI:** 10.1186/s12967-019-1907-2

**Published:** 2019-05-14

**Authors:** Clara Benna, Senthilkumar Rajendran, Marco Rastrelli, Simone Mocellin

**Affiliations:** 1 Department of Surgery Oncology and Gastroenterology, University of Padova, Padua, Italy; 2 Clinica Chirurgica I, Azienda Ospedaliera Padova, Padua, Italy; 3 Surgical Oncology Unit, Istituto Oncologico Veneto (IOV-IRCCS), Padua, Italy

**Keywords:** Sarcoma, Leiomyosarcoma, microRNA, miRNA, Pathway analysis, Dopamine, RNA polymerase III, RNA pol III

## Abstract

**Background:**

MicroRNA (miRNA) mediate post-transcriptional gene repression and are involved in a variety of human diseases, including cancer. Soft tissue sarcomas are rare malignancies with a variety of histological subtypes which may occur virtually anywhere in the human body. Leiomyosarcoma is one of the most common subtypes, shows a smooth muscle phenotype and its cancerogenesis is still unclear. The aim of our study was to investigate the potential role of miRNA differential expression in leiomyosarcoma development.

**Methods:**

We first employed the Sarcoma microRNA Expression Database, a repository that describes the patterns of over 1000 miRNA expression in various human sarcoma types, to identify differentially expressed miRNA comparing leiomyosarcoma and smooth muscle samples. Subsequently, we identified putative target genes of those miRNAs with the TargetScan prediction tool. Finally, we evaluated whether the retrieved pool of putative targets was enriched in genes belonging to specific molecular pathways by means of the Enrichr analysis tool. Protein–protein network analysis was analyzed by means of the STRING web tool.

**Results:**

Out of 1120 miRNAs tested, the expression of 301 miRNAs was statistically significantly different between leiomyosarcoma and smooth muscle samples. The hypothetical targets could be predicted for 172 miRNAs. 438 genes were predicted to be the targets with high confidence (cumulative weighted context score cut-off level less than − 1.0) and analyzed for belonging to specific molecular pathways. Pathway analysis suggested that RNA Polymerase III, tRNA functions and synaptic neurotransmission (with special regard to dopamine mediated signaling) could be involved in leiomyosarcoma development.

**Conclusions:**

Our results demonstrate that data mining of publicly available repositories can be useful to suggest molecular pathways underlying the pathogenesis of rare tumors such as leiomyosarcoma.

**Electronic supplementary material:**

The online version of this article (10.1186/s12967-019-1907-2) contains supplementary material, which is available to authorized users.

## Background

Leiomyosarcoma is the second most frequent type of soft tissue sarcoma (STS) accounting for approximately 14% of all cases and is defined as demonstrating a smooth muscle phenotype with immunohistochemistry [1, 2]. This rare tumor may occur virtually anywhere in the human body, from the limbs and trunk to the viscera and the retroperitoneum. Surgery remains the mainstay of treatment of localized leiomyosarcoma, but disease recurrence occurs frequently and chemotherapy is poorly effective to control metastatic disease [3]. Therefore, new therapies are eagerly needed in order to improve the prognosis of these patients. To this aim, understanding the cascade of molecular events leading to the development and progression of leiomyosarcoma plays a pivotal role in the identification of therapeutic targets.

miRNAs are 22–25 nucleotide RNAs that mediate post-transcriptional gene repression [4]. They are involved in a variety of human diseases, including malignancies, such as endometrial cancer [5], colorectal cancer [6], testicular cancer [7], acute myeloid leukemia [8], lung carcinoma [9], lymphoma [10], breast cancer [11] and osteosarcoma [12]. They can exert oncogenic functions as overexpressed miRNAs or serve as tumor suppressors and are consequently downregulated in the respective malignancy [13–15].

In this regard, recently, leiomyosarcoma has also been studied, moreover Authors’ attention has focused on miRNA signatures for their emerging potential as diagnostic biomarkers and for aiding subclassification [16–18], nevertheless the knowledge on this field of research is still poor.

The aim of our study is to further dissect molecular pathways involved in leiomyosarcoma development and progression. In a previous analysis [19] we explored the possible relation between the circadian clock pathway and soft tissue sarcoma (with special regard to leiomyosarcoma) susceptibility in terms of genetic variability. Here, we adopted a different strategy. First, we employed Sarcoma microRNA Expression Database (S-MED) https://www.oncomir.umn.edu/SMED/basic_search.php, a repository that describes the patterns of over 1000 miRNAs expression in various human sarcoma types, to individuate differentially expressed miRNA comparing leiomyosarcoma and smooth muscle tissues [20]. Subsequently, we identified putative target genes of those miRNAs with TargetScan prediction tool http://www.targetscan.org/ [21]. Finally, we evaluated whether the retrieved pool of putative targets was enriched in genes belonging to specific pathways http://amp.pharm.mssm.edu/Enrichr/ [22]. Finally, further insights in the interactions across gene products were evaluated by means of network analysis using the STRING web tool.

## Methods

### Study design

The objective of this study is to identify gene pathways whose expression is preferentially altered in leiomyosarcoma development. To achieve this goal we proceeded with the following 3 steps:

1. miRNAs finding: identification of differentially expressed miRNAs in leiomyosarcoma and smooth muscle tissues.
2. miRNAs to genes: identification of differentially expressed miRNAs target genes.
3. From genes to pathways: identification of gene pathways of differentially expressed miRNAs targets.

#### 1. miRNAs finding

Sarcoma-microRNA Expression Database (S-MED) [20] was employed to retrieve miRNA expression data in leiomyosarcoma and in smooth muscle. S-MED is a repository that describes the patterns of miRNA expression found in various human sarcoma tumor types and select normal tissues. S-MED provides both Basic and Advanced data search options for exploration of the data by means of heat-maps and text formats. Raw data were extracted for each of the considered miRNA. miRNAs in which the expression was not available in both leiomyosarcoma and smooth muscle were excluded. For each miRNA Student’s T-test was performed to assess significantly differentially expressed miRNAs between leiomyosarcoma and smooth muscle tissues. Bonferroni correction for multiple testing was applied to define the level of significance. P-values smaller than 4.50E−05 were considered significant.

#### 2. miRNAs to genes

TargetScan (v7.0; targetscan.org) [21] on line tool was employed for predicting effective microRNA target sites in human mRNAs. TargetScan predicts biological targets of miRNAs by searching for the presence of conserved 8mer, 7mer, and 6mer sites that match the seed region of each miRNA [23]. In mammals, predictions are ranked based on the predicted efficacy of targeting as calculated using cumulative weighted context++ scores of the sites [21]. The context++ score (CS) for a specific site is the sum of the contribution of 14 features [21]: site type, supplementary pairing, local AU, minimum distance, sRNA1A, sRNA1C, sRNA1G, sRNA8A, sRNA8C, sRNA8G, site8A, site8C, site8G, 3′ UTR length, SA, ORF length, ORF 8mer count, 3′ UTR offset 6mer count, TA (target site abundance), SPS (seed-pairing stability), P_CT_ (probability of conserved targeting). The cumulative weighted CS cut-off was set up at − 1.0 in order to minimize false positive associations between miRNA and their targets, as well as to yield a manageable number of targets to be considered with both enrichment and network analysis [24].

#### 3. From genes to pathways

Once target genes were identified, we used them to perform pathway analysis in order to identify biological functions whose genetic perturbations can predispose to leiomyosarcoma development.

For pathway analysis purposes, we utilized gene set enrichment analysis (GSEA) as performed by the EnrichR web server [22]. Hypergeometric distribution was used to calculate the statistical significance of gene overlapping [25], followed by correction for multiple hypotheses testing (using the false discovery rate [FDR] method) [26]. Only pathways with a FDR < 0.05 were considered of interest.

In contrast to pathways, networks are not based on specific biological functions but are built based on both direct (physical) and indirect (genetic) interactions between gene products (proteins). For network analysis, we utilized the STRING 11.0 web server [27]. The resulting network provides information of the degree of overall connectivity across imputed gene products (as quantified by the ratio between observed and expected interactions [a.k.a. “edges”] between proteins [a.k.a. “nodes”], and formally tested by means of a PPI enrichment test). Moreover, it suggests cluster of interacting proteins, which can help identify specific cell pathways.

## Results

### Differentially expressed miRNAs

**Fig. 1:**
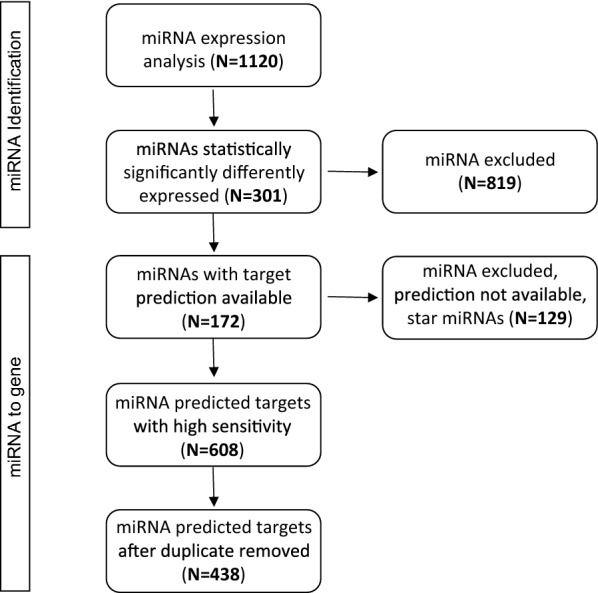
Flow diagram summarizing the miRNA search strategy and the gene targets selection process

The results are summarized in the flow chart in Fig. 1. In the first step of this study, we compared the expression between leiomyosarcoma and smooth muscle tissues of 1120 miRNAs whose expression data were stored in Sarcoma-microRNA Expression Database (S-MED). For each miRNA data were available for 32 samples (22 leiomyosarcoma tissues and 10 smooth muscle tissues). The expression of 301 miRNAs was statistically significantly different (P-value < 4.5 E−05) between neoplastic and normal tissues (see Additional file 1: Table S1). The hypothetical targets could be predicted for 172 miRNAs by the target prediction tool TargetScan. For 129 miRNAs, mostly star miRNAs (the complementary strand of the unstarred), target prediction was not possible: for this reason, those miRNAs were excluded from further analysis. Employing a cumulative weighted context score cut-off level of − 1.0 the retrieved putative gene targets were 608 (see Additional file 2: Table S2). After removing duplicates (which is because two or more miRNAs can share one or more target genes), the predicted targets resulted 438, which were considered for pathway analysis (see below section).

### From genes to pathways

**Fig. 2:**
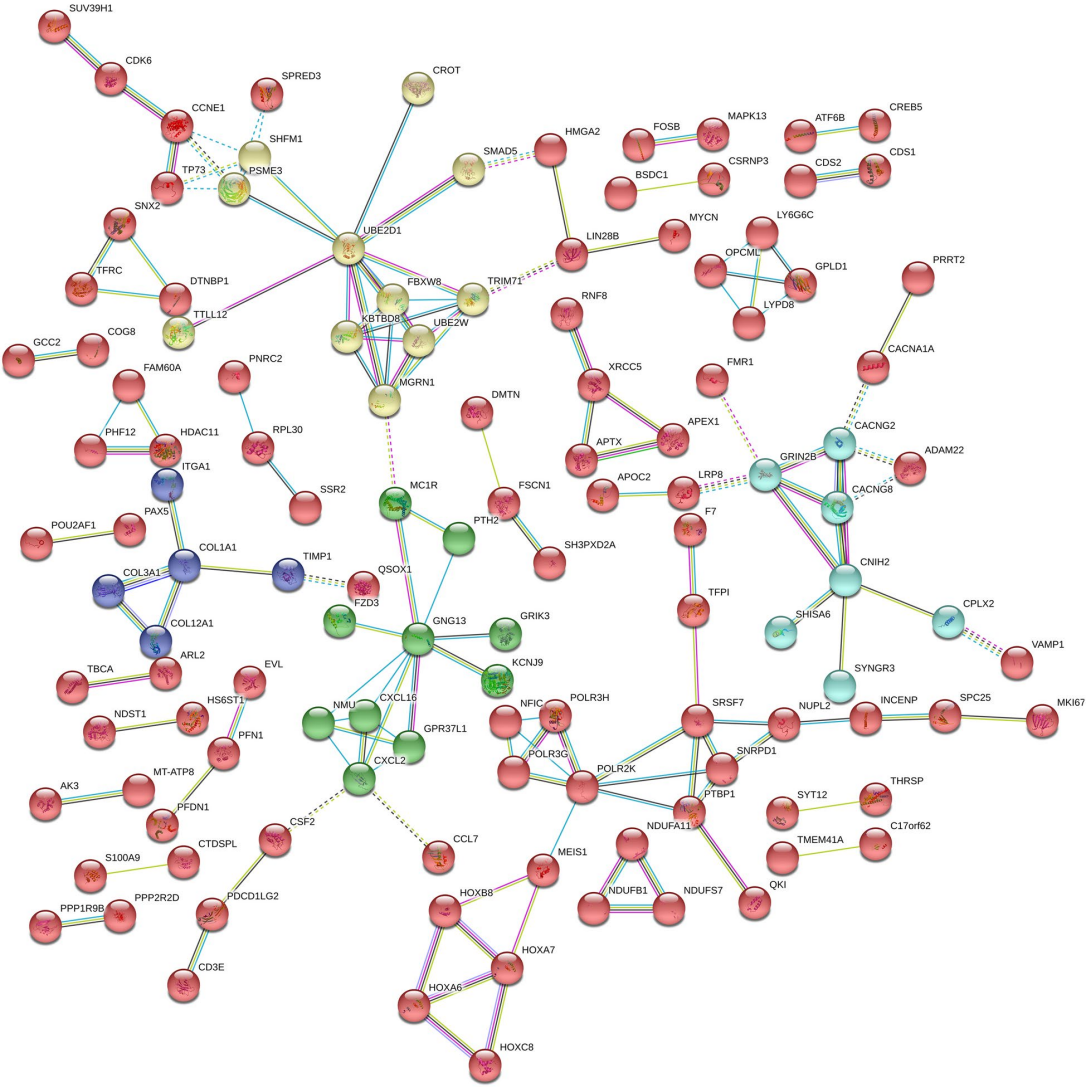
Network analysis of proteins encoded by putative miRNA target genes in leiomyosarcoma. The figure illustrates the high degree of connectivity of these proteins, which result to be involved in RNA pol III and tRNA functions and dopamine neurotransmission. Lines, light blue: from curated databases; purple: experimentally determined; light green: text mining; blue: gene co-occurrence; black: co-expression; light purple: protein homology

Primary gene set enrichment analysis suggested that the 438 putative miRNA targets are enriched in genes involved in RNA polymerase III and tRNA functions (as transcription initiation, elongation, termination and tRNA modification) and in synaptic neurotransmission. Interrogating the KEGG and Reactome databases, the EnrichR webtool returned the results reported in Table 1. Network analysis of proteins encoded by miRNA targets showed that the overall connectivity was significantly greater than expected (144/122 edges, P = 0.03) and confirmed the results obtained by enrichment analysis, especially underscoring the high number of interactions between proteins involved in neurotransmission (see Fig. 2).

**Table 1:** Pathway analysis main findings: gene set enrichment analysis based on 438 miRNA target genes

Pathway	Overlap	FDR	Genes	Database
RNA polymerase III functions				
RNA polymerase III transcription termination	4/23	0.0014	NFIC; POLR3G; POLR3H; POLR2K	REACTOME
RNA polymerase III chain elongation	3/18	0.0067	POLR3G; POLR3H; POLR2K	REACTOME
tRNA modification in the nucleus and cytosol	4/39	0.0102	URM1; ADAT1; LCMT2; OSGEP	REACTOME
RNA polymerase III transcription	4/41	0.0121	NFIC; POLR3G; POLR3H; POLR2K	REACTOME
RNA polymerase III	3/32	0.0324	POLR3G; POLR3H; POLR2K	KEGG
Synaptic neurotransmission				
Dopaminergic synapse	9/129	0.0022	KCNJ6; ATF6B; KCNJ9; PPP2R2D; CACNA1A; GRIN2B; CREB5; GNG13; MAPK13	KEGG
Synapse formation and maturation (LGI-ADAM interactions)	3/14	0.0032	CACNG8; ADAM22; CACNG2	REACTOME
Depolarization of the presynaptic terminal triggers the opening of calcium channels	2/13	0.0318	CACNA1A; CACNG2	REACTOME
Neurotransmitter receptor binding and downstream transmission in the postsynaptic cell	7/142	0.0370	CACNG8; KCNJ6; KCNJ9; GRIK3; CACNG2; GRIN2B; GNG13	REACTOME
Morphine addiction	5/91	0.0496	KCNJ6; KCNJ9; CACNA1A; GABRG1; GNG13	KEGG
Other pathways				
Extrinsic pathway of fibrin clot formation	2/5	0.0046	F7; TFPI	REACTOME
Chemokine receptors bind chemokines	5/56	0.0075	CCL22; CCL7; ACKR2; CXCL2; CXCL16	REACTOME
TP53 regulates transcription of genes involved in G1 cell cycle arrest	2/13	0.0318	CCNE1; ZNF385A	REACTOME
Regulation of TP53 activity through association with co-factors	2/14	0.0366	ZNF385A; TP73	REACTOME
Hematopoietic cell lineage	5/88	0.0440	FCER2; CSF2; TFRC; ITGA1; CD3E	KEGG
Protein digestion and absorption	5/90	0.0477	COL1A1; SLC6A19; COL3A1; COL12A1; SLC8A2	KEGG

*FDR* false discovery rate

## Discussion

The aim of our study was to identify molecular pathways whose expression is particularly affected during leiomyosarcoma development. Our approach was first to identify miRNA which are statistically significantly down or up-regulated in leiomyosarcoma compared to smooth muscle, second to predict putative targets of those miRNA, and third to analyze if those putative target genes belonged to specific molecular pathways. Our results suggest that RNA Polymerase III, tRNA functions and synaptic neurotransmission could be altered during leiomyosarcoma development.

### RNA polymerase III, tRNA and cancer

RNA polymerase III (pol III) is the largest RNA polymerase with the greatest number of subunits. It synthesizes a range of essential products, including tRNA, 5S rRNA and 7SL RNA, which are required for protein synthesis and trafficking. Moreover, while RNA polymerase I (pol I) synthesizes three ribosome subunits as a single precursor transcript that is processed into the final mature products, pol III produces MPR RNA, necessary for the processing; for a review see [28, 29]. Abnormal pol III activity has been proposed to be feature of cancer cells. rRNA and tRNA are overproduced consistently in different human cancers as ovarian [30] breast, lung and tongue carcinomas [31, 32]. Conversely, in healthy cells oncogenes and tumor suppressor signaling pathways, such as the PI3kinase/TORC1, Ras/ERK, Myc, p53 and Rb pathways, regulate Pol III and tRNA synthesis. In particular, pol III transcription factor (TFIIIB) interacts with the tumor suppressors RB and p53 to limit pol III production [33, 34]. Regarding tRNA, a pathway not related to pol III has also been identified in our study, which is tRNA modification in the nucleus and cytosol. Wobble tRNA modifications are required during translation elongation and sustain proteome homeostasis. A recent work has highlighted the upregulation of the wobble uridine 34 (U34) tRNA cascade in cancer, which underlies the specific requirement for this pathway in tumor development [35]. It is plausible to suppose that a further mechanism of controlling pol III abundance and tRNA production and maturation is due to miRNAs. Our results support this hypothesis and suggest that evading this form of control could contribute to leiomyosarcoma development.

### Dopamine

Dopamine is a monoamine neurotransmitter, synthesized from the amino acid tyrosine, which is transported from the liver to the brain via an active transport mechanism. Dopamine plays central role in pleasurable reward behavior, hormone secretion, sleep, mood, attention, learning, behavior, control of nausea and vomiting, and pain processing.

Due to extensive localization of dopamine receptor to brain areas and its role in wide range of functions, dopaminergic dysfunction has been implicated in the pathophysiology of mood disorders, schizophrenia, obsessive compulsive disorder, autism spectrum disorders, attention deficit–hyperactivity disorder, Tourette’s syndrome, substance dependency, Parkinson’s disease and other disorders [36, 37].

Dopamine receptors (DRs) belong to the family of seven transmembrane domain G-protein coupled receptors and are classified into D1-like (DR1) and D2-like receptor families (DR2) based on pharmacological properties, structure, and signal transduction system. DRs subtypes are expressed not only in brain areas, but also in many tissues and organs as kidney, heart, and the peripheral nervous. For this reason, this catecholamine also modulates cardiovascular function, vascular tone, renal function, and gastrointestinal motility.

### Dopamine and smooth muscle

In recent works, many Authors found that the dopamine DR1 receptors are expressed on airway smooth muscle and regulate smooth muscle force via cAMP activation of PKA [38]. Moreover, it is reported that DR1 activation inhibited proliferation of the vascular smooth muscle cells [39].

### Dopamine and osteosarcoma

Gao et al. [40] results suggest that DR1 are expressed in the osteosarcoma cells and inhibit the proliferation of osteosarcoma cells by the down-regulation of the ERK1/2 and PI3K-Akt pathways. In a different study, the same research group [41] suggested that activation of DR1 induces osteosarcoma cell apoptosis via changes to the MAPK pathway. The Authors proposed DR1 as a novel target for the treatment of osteosarcoma.

Here, the results of the present study support the hypothesis that dopamine pathway is involved in sarcoma growth and development and in particular in leiomyosarcoma.

### Potassium voltage-gated channel and cancer

Considering each of the single target genes listed in this pathway, we found two potassium voltage-gated channel: Potassium Voltage-Gated Channel Subfamily J Member 6 (KCNJ6) and Potassium Voltage-Gated Channel Subfamily J Member 9 (KCNJ9). The ultimate effect of DR1 can be excitation (via opening of sodium channels) or inhibition (via opening of potassium channels). Voltage-gated potassium channels (Kv), encoded by 40 genes in humans, are the largest subset of potassium channels gated by changes in the membrane potential [42]. Numerous studies have reported dysregulated potassium channel expression in human cancer [43]. In particular, few Authors focused on sarcoma: a study showed that that Kv1.3 voltage-gated potassium channels was upregulated in human osteosarcoma and downregulation of Kv1.3 suppressed osteosarcoma growth in vivo and osteosarcoma cell proliferation in vitro, accompanied by increased apoptosis [44]. Similar results were shown for the voltage-gated potassium channels Ether à go-go 1 and Kv1.5 [45, 46].

## Limitations

This study provides useful insights for further studies, nevertheless it is limited by a number of weaknesses. The initial analysis was carried out on 1120 miRNAs for which data were available on SMED: nevertheless, identified human miRNA are at least twice as much (as reported by international databases such as MirBase, http://www.mirbase.org/), which leaves room for many more miRNAs to be evaluated in leiomyosarcoma pathogenesis. In addition, we could retrieve hypothetical target genes only for a miRNA subset because the TargetScan repository does not report data on star miRNAs, which, although usually degraded, sometimes also function as gene expression regulators [47, 48]. Moreover, the analyses reported by the SMED web tool were based on 22 cases and 10 control samples: this small sample size does not protect against both false positive and false negative association results. As regards the target genes we have identified as potentially targeted by miRNA specifically deregulated in leiomyosarcoma, we could not validate our results in silico due to the lack of publicly available data on leiomyosarcoma. Consequently, dedicated experiments on human leiomyosarcoma samples are necessary to verify our hypothesis. Finally, though our results might suggest new therapeutic targets in the fight against leiomyosarcoma, experimental evidence proving the efficacy of this strategy is obviously warranted.

## Conclusions

In soft tissue sarcomas, various miRNAs are differentially expressed, supporting the hypothesis that they could contribute to development, progression and invasion of this rare group of malignancies. Here, we focused on leiomyosarcoma, one of the most frequent types of soft tissue sarcomas. Overall our results suggest that there are pathways particularly targeted by miRNA altered expression which are already been linked to other malignancies, but are worth to be studied in leiomyosarcoma such as RNA pol III and tRNA functions and dopamine neurotransmission pathway. Expression and functional studies are needed to ascertain the role of these specific pathways in leiomyosarcoma development.

In general, our results demonstrate that data mining of publicly available repositories can be useful to suggest molecular pathways underlying the pathogenesis of rare tumors such as leiomyosarcoma.

## Additional files

**Additional file 1: Table S1.** miRNAs retrieved in S-MED (Sarcoma-microRNA Expression Database), Student T-test P-val. Red: miRNAs with available targets prediction in TargetScan.

**Additional file 2: Table S2.** miRNAs targets predicted by TargetScan with cumulative weighted context score cut-off level of − 1.0 or smaller.

## Data Availability

All data generated or analysed during this study are included in this published article and its Additional files.

## Abbreviations

miRNA: microRNA
STS: soft tissue sarcoma
S-MED: Sarcoma microRNA Expression Database
CS: context++ score
GSEA: gene set enrichment analysis
FDR: false discovery rate
pol III: RNA polymerase III
pol I: RNA polymerase I
TFIII: pol III transcription factor
DRs: dopamine receptors
DR1: D1-like receptor family
DR2: D2-like receptor family
